# Accidental Pyrethroid Ingestion in Toddler: Near-Fatal Atypical Presentation and Successful Recovery

**DOI:** 10.3389/fped.2019.00542

**Published:** 2020-01-10

**Authors:** Marco Pallavidino, Diego Arango Uribe, Subashini Baskaran, Aqdas Saqib, Mohamed Elmesserey, Ahmed Onsy, Emad M. Fathi, Christoph Fink, Anil Kumar H. Ramaiah

**Affiliations:** Department of Paediatric Intensive Care Medicine, Al Jalila Children's Speciality Hospital, Dubai, United Arab Emirates

**Keywords:** pyrethroid, ingestion, transfluthrin, children, intoxication

## Abstract

We are reporting a case of pyrethroid poisoning with atypical presentation in a 21-month-old toddler who was transferred to us from a peripheral center. Signs and symptoms at presentation were predominantly of cardiopulmonary dysfunction contrary to more common presenting features of gastrointestinal and neurological impairment. The reason for this seems to be the aspiration pneumonitis as a consequence of vomiting induced by parents at home, rather than the toxin itself even though a rather rapid progression of lung injury does not rule out the possibility. He had developed decreased level of consciousness and increased work of breathing after ingestion, which had progressed to Acute Respiratory Distress Syndrome, septic shock, and multi organ failure. He even had a brief cardiac arrest with Return of Spontaneous Circulation after 5 min of cardiopulmonary resuscitation, immediately after arrival at our unit, which seemed more likely to be a consequence of inappropriate management during transfer of the child. In addition to antibiotics and vasopressors, he required high frequency oscillatory ventilation and prone positioning initially, and lung-protective conventional ventilation later. His cardiopulmonary status improved gradually and he was successfully extubated after 12 days. Other organ systems also showed complete recovery. Even though Magnetic Resonance Imaging of brain done a few days after cardiac arrest showed features suggestive of hypoxic-ischemic encephalopathy he showed complete neurological recovery. He was thriving well at three-month follow-up with no neurological deficits, good exercise tolerance, and normal renal and liver function. Atypical presentation of pyrethroid poisoning is associated with significant morbidities and there seems to no reliable parameters in children to identify the risk of the same. Considering that there is no specific antidote, prompt, and aggressive supportive therapy is necessary for a favorable outcome. This case highlights several important aspects in the care of the pediatric patient after ingestion of insecticides. First, attempt to induce emesis, especially outside of a healthcare facility is not only ineffective but also highly dangerous, and should not be done. Second, unstable patients require inter and intrahospital transfer by experienced and trained personnel; and lastly, management for these complex and atypical cases should be done as early as possible in a center which is equipped to provide high level of circulatory and ventilatory support while prioritizing neuro-protective measures, and neurologic recovery and rehabilitation.

Transfluthrin is a fast acting synthetic pyrethroid insecticide. It is used in household-hygiene products, mainly against flying insects, such as mosquitoes and flies, and material pests, such as moths (1). Oral ingestion is the most common route of intoxication and mainly causes central nervous system (CNS) symptoms like headache, dizziness, drowsiness, status epilepticus, and respiratory failure. Pyrethroids act by prolonging the opening of sodium channels leading to increased influx of sodium ions causing hyper-excitation of the nervous system (2). Even though cardiac dysfunction as a result of toxicity has been described, lung injury is not a common feature (3, 4). There is no specific antidote for transfluthrin toxicity and management is mainly symptomatic and supportive (5). We report a case of transfluthrin toxicity in a 21 month child who had an atypical presentation with predominant features of lung injury.

## Case Report

A 21-month-old toddler was referred to us with a history of ingestion of about 2–4 ml of a mosquito repellent containing Transfluthrin. Parents had attempted to induce vomiting and had taken him to a nearby hospital immediately after. His level of consciousness had started decreasing and reached a Glasgow Coma Scale (GCS) of 11, along with increased work of breathing. He had developed an episode of seizure which was treated with Phenobarbital and Levetiracetam. Though his neurological status improved in the first 24 h itself, he continued to have respiratory distress which progressed to acute respiratory distress syndrome (ARDS) over the next 72 h and had to be started on lung protective, invasive mechanical ventilation. He also needed vasopressors since he had become unstable hemodynamically. As the clinical condition was not improving and the referring hospital did not have a Pediatric intensive Care Unit he was transferred to our hospital.

**Figure 1:**
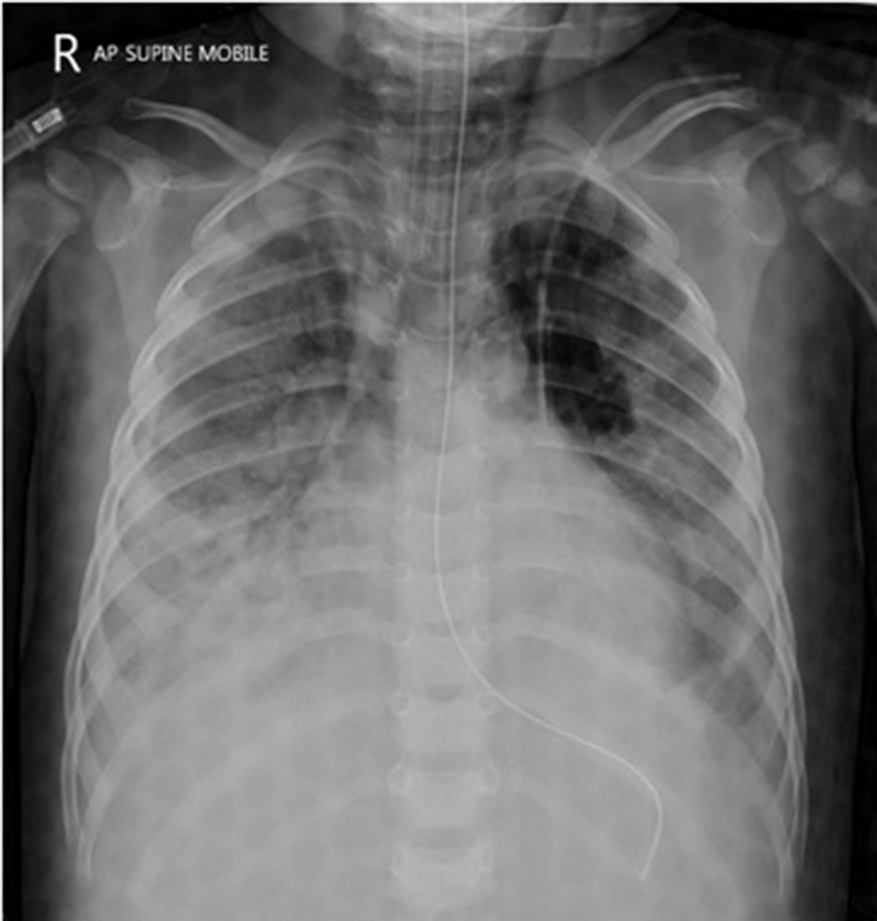
Chest X Ray at PICU admission (48 h after the ingestion).

**Figure 2:**
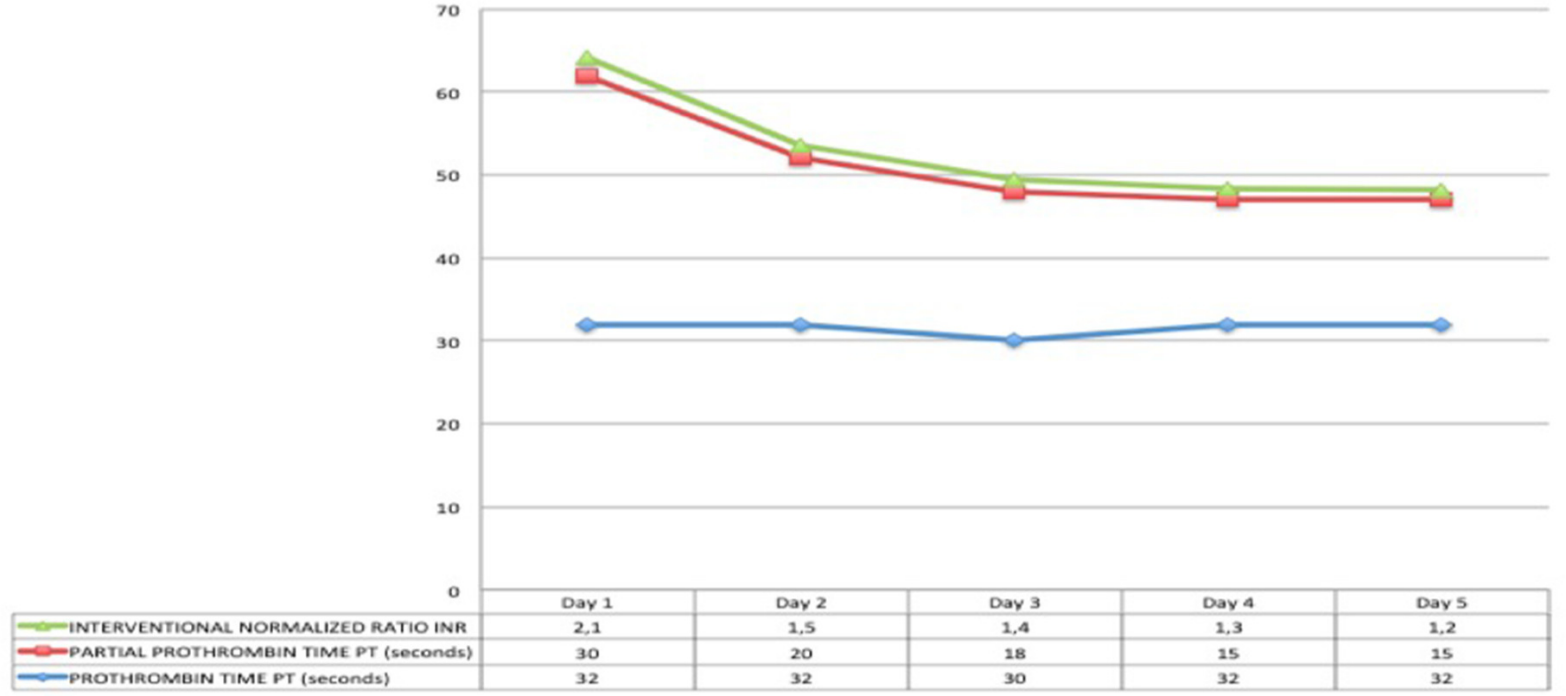
Trend of Coagulation markers (Day 1–5).

**Figure 3:**
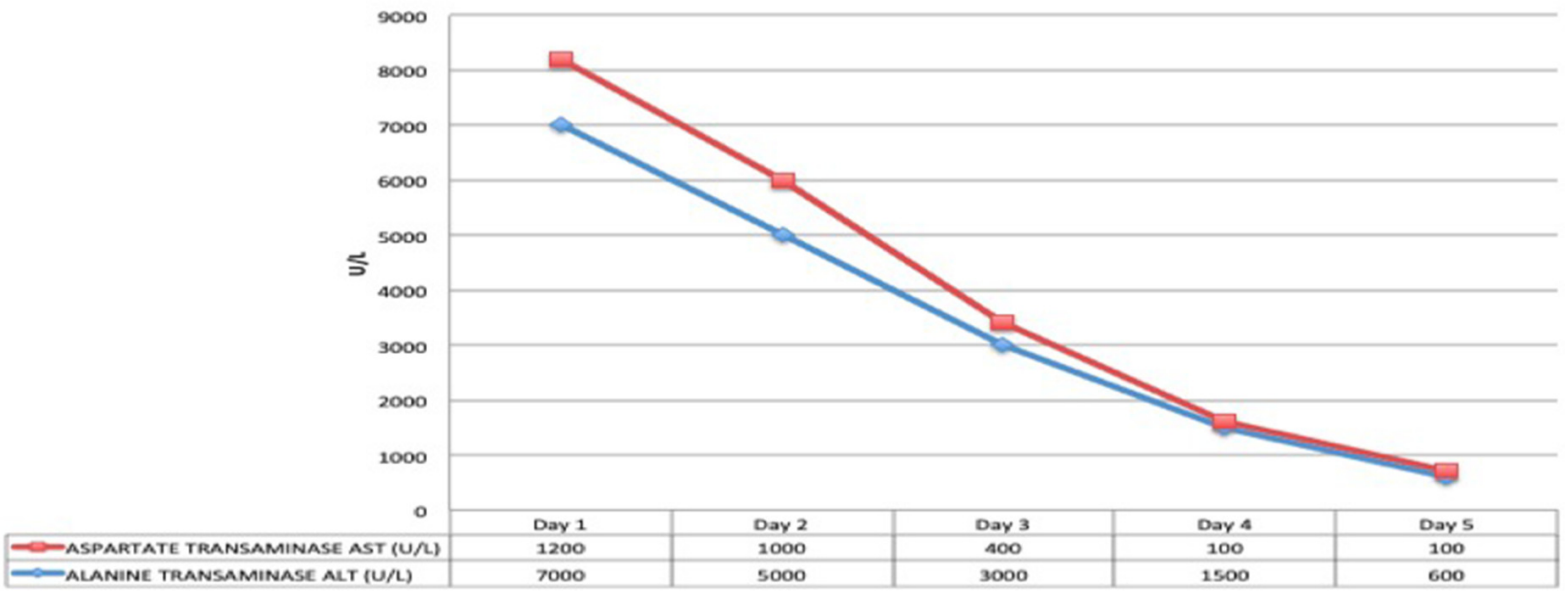
Trend of liver function markers (Day 1–5).

At the time of arrival on our unit, he was severely hypoxic, most probably because of suboptimal ventilatory support during transfer despite sedation and paralysis. With a few minutes, he had a cardiac arrest with return of spontaneous circulation after 5 min of cardiopulmonary resuscitation. Initial investigations showed diffuse, bilateral pulmonary opacities on chest X-ray (Figure 1), ratio of partial pressure of oxygen in arterial blood/fractional oxygen concentration (P_a_O_2_/FiO_2_) of 65, and oxygenation index (OI) of 41. He was started on high frequency oscillatory ventilation at FiO_2_ of 100% along with nitric oxide, and was nursed in prone position. His oxygenation improved with these measures even though he remained hemodynamically unstable with features of septic shock requiring high doses of norepinephrine and epinephrine. Infection markers were raised, with procalcitonin of 65 ng/ml, white blood cell count of 16,000/μl, and neutrophils of 75%. Antibiotics were empirically changed to vancomycin and meropenem after taking pan cultures which eventually came back negative for microbial growth. Both hemodynamic status and sepsis markers improved over next 24 h (Figure 2). There was fall in serum lactate levels from 9 mmol/L to 1.9 and good urine output suggesting improved perfusion. He had significant liver injury on admission with Alanine Transaminase (ALT) and Aspartate Transaminase (AST) of >7,000 and 4,320 IU/L, respectively, along with periportal hypoechogenicity on ultrasound (Figure 3). These resolved gradually over the first 6 days of admission. Enteral feeding was started after 48 h of admission and was well-tolerated. There was no significant acute kidney injury anytime and he had good urine output once the hemodynamic status improved. Vasopressors were weaned off gradually and he was changed to conventional ventilation 72 h after admission. Ventilatory support was gradually weaned and he was successfully extubated 12 days later.

MRI was done once the child was hemodynamically stable on PICU, which showed features of hypoxic-ischemic encephalopathy. He was monitored daily with transcranial Doppler and no abnormalities like signs of midline shift, the Diastolic Flow Velocity, Pulsatility Index and Resistance Index were detected any time.

**Figure 4:**
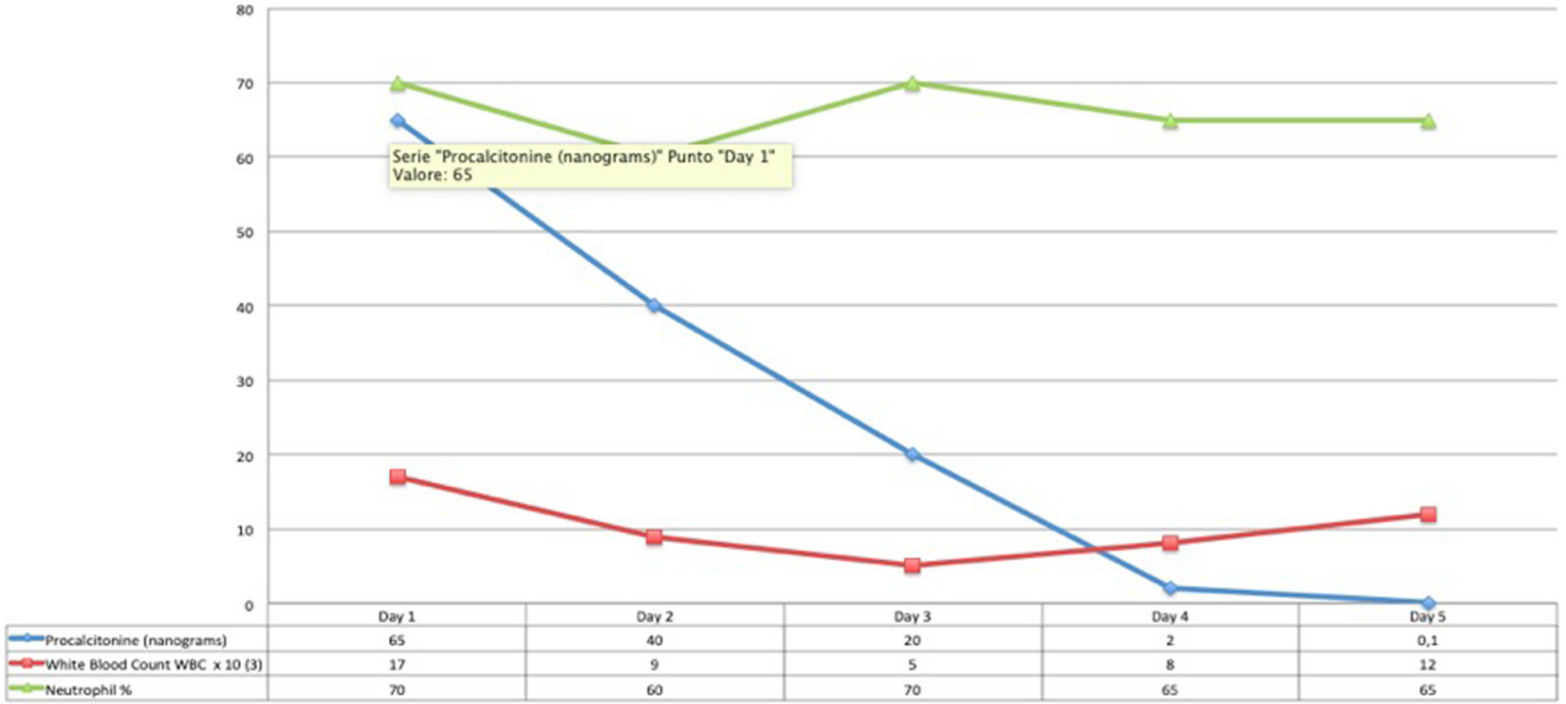
Trend of infection markers (Day 1–5).

He had developed ischemic necrosis in his right foot following arterial line insertion in right femoral artery. This was treated with heparin infusion, glyceryl trinitrate patches, and eventually debridement (Figure 4).

He had a total ICU stay of 21 days and hospital stay of 25 days. He was thriving well at 3 month follow-up with no neurological deficits, good exercise tolerance, and normal renal and liver functions.

## Discussion

Pyrethroids are widely-used, multipurpose insecticides with a long safety record for both acute and chronic intoxication in human beings. They act by prolonging the activation of the neuronal voltage-dependent Na+ channel by binding to the channel's open state causing prolonged depolarization. Since, the mammalian voltage-dependent sodium channel, unlike its insect counterpart, has many isoforms, mammals are relatively resistant to its actions (9). They are about 2,250 times more toxic to insects compared with mammals. Therefore, toxicity syndrome due to accidental ingestion is uncommon and even more so in children (8). The absence of a specific antidote makes the management of intoxication challenging and aggressive symptomatic management seems like the best course of action.

Most patients with pyrethroid poisoning recover within 6 days and fatality appears infrequent. There were seven fatalities among 573 cases in one series and one among 48 in another (6, 7). Out of the 573 cases of acute pyrethroid poisoning reported by He et al., four of seven fatalities were due to convulsions and one was due to pulmonary edema (7).

Atypical presentation of the poisoning has been defined in an earlier report as patients with respiratory failure requiring mechanical ventilation, hypotension (systolic blood pressure < 90 mm Hg), pneumonia, acute kidney injury (AKI, Cr > 1.4 mg/dL), seizure, GCS < 15, and death (9). Our case had an atypical presentation of pyrethroid poisoning with predominantly cardiopulmonary dysfunction contrary to the more common presenting features of gastrointestinal and neurological impairment. The reason for this seems to be the aspiration pneumonitis as a consequence of vomiting induced by parents at home. The rather rapid deterioration of his ARDS to an OI of 41, raises the possibility of direct toxin effect on the lung parenchyma. A similar event has been reported by Peter et al., which unfortunately ended in a fatality, due to aspiration pneumonia within 10 h after ingestion of 30 ml of cypermethrin (12). This underlines the fact that best course of action after intoxication is treatment at the hospital and home remedies should not be attempted.

Invariably these children would need care in the critical care unit and there might be need for interhospital transfer because of this. Adverse events and deterioration during interhospital, and even intrahospital, transfer is highly likely if a trained team is not involved in the process (10, 11). Our patient arrived to our unit in an extremely critical condition and according to the referring physician much of the deterioration happened during transfer. So, it is important to have a trained regional transport team and they should do transfers in all critically ill children. Also, it is crucial to refer these children sooner than later to a tertiary care facility as they may need high level of organ support.

Pyrethroids are relatively safe insecticides and have been in use for a long time. However, atypical presentation of poisoning is associated with significant morbidities, and even mortality. Even though, it has been reported earlier that if the amount ingested > 250 cc and serum lactate is >3.5 mmol/L at presentation, could be predictors of atypical presentation in adults, we are not sure we extrapolate these values for children. Considering that there is no specific antidote, aggressive supportive therapy is necessary for a favorable outcome, preferably in a center which is equipped to provide high level of circulatory and ventilatory support.

## Data Availability Statement

The datasets generated for this study are available on request to the corresponding author.

## Ethics Statement

Ethical review and approval was not required for the study on human participants in accordance with the local legislation and institutional requirements. Written informed consent to participate in this study was provided by the participants' legal guardian/next of kin. Written informed consent was obtained from the individual(s), and minor(s)' legal guardian/next of kin, for the publication of any potentially identifiable images or data included in this article.

## Author Contributions

All authors contributed to manuscript revision, read, and approved the submitted version.

### Conflict of Interest

The authors declare that the research was conducted in the absence of any commercial or financial relationships that could be construed as a potential conflict of interest.
